# Recurrent neural network architecture for forecasting banana prices in Gujarat, India

**DOI:** 10.1371/journal.pone.0275702

**Published:** 2023-06-15

**Authors:** Prity Kumari, Viniya Goswami, Harshith N., R. S. Pundir

**Affiliations:** 1 College of Horticulture, Anand Agricultural University, Anand, Gujarat, India; 2 B. A. College of Agriculture, Anand Agricultural University, Anand, Gujarat, India; 3 IABMI, Anand Agricultural University, Anand, Gujarat, India; Cairo University, EGYPT

## Abstract

**Objectives:**

The forecasting of horticulture commodity prices, such as bananas, has wide-ranging impacts on farmers, traders and end-users. The considerable volatility in horticultural commodities pricing estimates has allowed farmers to exploit various local marketplaces for profitable sales of their farm produce. Despite the demonstrated efficacy of machine learning models as a suitable substitute for conventional statistical approaches, their application for price forecasting in the context of Indian horticulture remains an area of contention. Past attempts to forecast agricultural commodity prices have relied on a wide variety of statistical models, each of which comes with its own set of limitations.

**Methods:**

Although machine learning models have emerged as formidable alternatives to more conventional statistical methods, there is still reluctance to use them for the purpose of predicting prices in India. In the present investigation, we have analysed and compared the efficacy of a variety of statistical and machine learning models in order to get accurate price forecast. Autoregressive Integrated Moving Average (ARIMA), Seasonal Autoregressive Integrated Moving Average model (SARIMA), Autoregressive Conditional Heteroscedasticity model (ARCH), Generalized Autoregressive Conditional Heteroscedasticity model (GARCH), Artificial Neural Network (ANN) and Recurrent Neural Network (RNN) were fitted to generate reliable predictions of prices of banana in Gujarat, India from January 2009 to December 2019.

**Results:**

Empirical comparisons have been made between the predictive accuracy of different machine learning (ML) models and the typical stochastic model and it is observed that ML approaches, especially RNN, surpassed all other models in the majority of situations. Mean Absolute Percent Error (MAPE), Root Mean Square Error (RMSE), symmetric mean absolute percentage error (SMAPE), mean absolute scaled error (MASE) and mean directional accuracy (MDA) are used to illustrate the superiority of the models and RNN resulted least in terms of all error accuracy measures.

**Conclusions:**

RNN outperforms other models in this study for predicting accurate prices when compared to various statistical and machine learning techniques. The accuracy of other methodologies like ARIMA, SARIMA, ARCH GARCH, and ANN falls short of expectations.

## Introduction

The Indian economy is heavily reliant on the agricultural sector. More than 70% of rural households are dependent on agriculture. Given that it accounts for 20% of the country’s GDP and employs more than 60% of the workforce, India’s agricultural industry plays a crucial role in the nation’s economy. Over the past few years, Indian agriculture has experienced significant growth [1]. Processing, floriculture, seed production, mushroom cultivation, nursery preparation, post-harvest production etc., all fall within the horticultural umbrella, making it an essential and crucial component of stabilising farmers’ income [2]. The scenario of horticultural crops in India has become very encouraging. During 2020, according to the National Horticulture Board (NHB) [3], the production of horticultural crops in India was 320.77 million tonnes from an area of 26.46 million hectares. It is further expected to grow at the rate of 1.8% in financial year 2021 with the area expected at 27.17 million hectares and production 326.58 million tonnes.

Bananas are widely grown in the tropics and subtropics. It is the world’s seventh most traded agricultural product, behind wheat, maize, soybeans, rice, barley, and sugar [4]. Annually, 14.2 million tonnes of banana are produced in India, which is more than any other country in the world. After the mango, the banana is India’s second most popular fruit. Tamil Nadu, Andhra Pradesh, Maharashtra, and Karnataka contribute to a significant percentage of banana production in India [5]. Bananas have long been revered for their many cultural, medical and dietary benefits. It is great source of carbohydrates (22.84 g/100 g), provides around 370 kJ of energy per 100 grams and also considered as one of the best sources of potassium (358 mg/100 g) that provides 8 percent of the recommended daily intake [6]. India is the world’s largest producer and exporter of bananas with an estimated 884 thousand hectares under cultivation and production of about 30 million tonnes (MT), contributing around 26% to the global banana basket. India holds a significant position among the top five banana-producing countries, accounting for approximately 50% of the total share (FAOSTAT 2019) [7]. Additionally, Gujarat, as the second-ranked state in banana production within India, contributes approximately 24% of the total share among the top five Indian states, as reported by the National Horticulture Board (NHB) [3].

In Gujarat, the area under production is 69.537 thousand hectares with production 4627.52 metric tonnes (Directorate of Horticulture, 2021) [8]. According to market arrival statistics, the Rajpipla market (Narmada) accounts for 78% of Gujarat’s overall arrival, making it the state’s primary market for determining banana prices(Agriculture marketing website 2020) [9].

Naturally, anticipating what will happen next, rather than why something happened in the past, is one of the primary purposes of time series analysis. One can use a forecasting model based on the experience and external information. Auto Regressive Integrated Moving Average (ARIMA) is most widely used time-series models amongst the base statistical models. Recently, the Artificial Neural Network (ANN) model has gained much of attention as a potential replacement of traditional models for estimation and forecasting in economics and finances [10].

Future price predictions are exceedingly difficult to make. There is a substantial body of literature on various techniques and predictors that can be added to those techniques in order to achieve higher accuracy [11]. Farmers will be able to utilise this information to determine the best time to sell their crops. As a result, the frequency and accessibility of time- and location-based arbitrage should reduce the volatility of prices [12].One of the biggest obstacles to making reliable banana price predictions is the seasonality of banana price series. Given the complexity of the price series, several models have been established for capturing price behaviour, but there is no consensus between researchers as to which model is best [13].

Numerous linear and nonlinear methods have been developed within the time series framework, including the Autoregressive Integrated Moving Average (ARIMA) model, the Seasonal ARIMA (SARIMA) model and the Generalized Autoregressive Conditional Heteroscedastic (GARCH) model. Multiple previous studies have attempted to predict agricultural commodity prices [14–18]. The SARIMA model is supposedly superior to other price forecasting algorithms when it comes to predicting onion prices in Mumbai’s marketplaces [14]. Application of the ARIMA model for forecasting agricultural productivity in India can be found in [15]. Application of the SARIMA model for forecasting meat exports from India [16]. Price volatility for agricultural commodities in India has been extensively studied in [17]. Spot electricity price forecasting in Indian electricity market using autoregressive-GARCH models can be seen in [18].

Machine Learning (ML) methods that have recently emerged under the data science [19] has become dominant approach of modelling. Time series forecasting, in the fields of finance and economics, have greatly benefitted by its application [20, 21] and also has been applied to forecast the area, production and productivity of agricultural commodities like citrus [22], banana [23] and mango [24]. Several empirical studies have demonstrated that when forecasting various assets, ML algorithms outperform time series models [25–27]. Comparison of efficacy of statistical models and machine learning techniques can be found in [28]. It is reported that ANNs works well over the classical statistical methods such as linear regression and Box- Jenkins approaches [29]. For both price and yield forecasting in agriculture, neural networks were shown to be more accurate than statistical techniques[30, 31]. Superiority of neural network in price forecasting [32], in percent losses of pods by pod borer and pod fly in pigeon pea [33], were discussed. While application of RNN model found in forecasting prices of arecanuts in Kerala [34] and in agricultural product prices prediction [35].

The primary goal of this study is to forecast the price of banana in Gujarat, India using ARIMA, SARIMA, ARCH, GARCH, ANN and RNN by using secondary data obtained from the Agricultural Marketing website, focusing on banana prices in the major market of Gujarat covering the period from January 2009 to December 2019. The research aims to identify the forecasting model that exhibits the highest level of prediction accuracy, assessed through performance metrics such as RMSE, MAPE, SMAPE, MASE and MAD.

## Materials and methods

The sample consisted of 132 observations (i.e., monthly data for 11 years). The data represents the modal prices of banana. One important characteristic of the dataset used in this investigation is that it spans over 11 years, which is helpful in analysing long-term trends and patterns in the market. However, the dataset has its own limitations of having missing observations at long length apart from potential errors in the data collection.

Following analytical models were utilized in the present study:

### Statistical models

#### ARIMA—Auto Regressive Integrated Moving Average model

George Edward Pelham Box and Gwilym Meirion Jenkins proposed the ARIMA (p, d, q) model in the 1970s which is called Box-Jenkins model [36]. In the ARMA model, a variable’s projected future value is a linear mixture of its previous lag value and error:

$$y_{t}=\emptyset_{1}y_{t-1}+\emptyset_{2}y_{t-2}+\ldots +\emptyset_{p}y_{t-p}+\varepsilon_{t}-\emptyset_{1}\varepsilon_{t-1}-\emptyset_{2}\varepsilon_{t-2}\ldots -\emptyset_{p}\varepsilon_{t-p}$$ (1)

where, *y*_*t*_ is the actual value at t, {*ε*_*t*_} is the white noise sequence, *p and q* are integers which are called autoregressive and moving average, respectively. When dealing with a non-stationary time series, difference can be made to make it stationary series.

#### SARIMA—Seasonal Auto Regressive Integrated Moving Average model

Box-Jenkins generalized this model to deal with seasonality. This model uses seasonal differencing to remove non-stationarity.

SARIMA (p, d, q) * (P, D, Q)^S^ is presented below in terms of Lag polynomial:

$$\Phi_{P}(L^{S})\varphi_{P}(L){\left(1-L\right)}^{d}{\left(1-L^{S}\right)}^{D}y_{t}=\theta (L^{S})\theta_{q}(L)\varepsilon_{t}$$ (2)

Here *L*, *L*^*S*^ are non-seasonal and seasonal differencing, *φ*_*P*_ *(L) & θ*_*q*_ *(L)* are parameters of *“p”* and *“q”* for non-seasonal lag value, *Φ*_*P*_*(L*^*S*^*) and Θ(L*^*S*^*)* are parameters of *“P” and “Q”* for seasonal lag while *y*_*t*_ and *ε*_*t*_ are original time series and error at given time t [37].

#### ARCH—Autoregressive Conditional Heteroscedasticity model

Autoregressive conditional heteroscedasticity (ARCH) models predict conditional variances. The ARCH model comprises two primary components: the mean equation and the variance equation. The mean equation specifies the conditional mean of the series and typically incorporates autoregressive or moving average terms. On the other hand, the variance equation models the conditional variance of the series. Engle (1982) [38] first suggested ARCH models, later generalised as GARCH [39].

#### Generalized Autoregressive Conditional Heteroscedasticity (GARH) models

GARCH models offers enhanced capabilities in capturing the intricate dynamics of volatility. Then GARCH model (p, q) by Bera, A. K. & Higgins, M. L. (1993) [40] is:

$$\sigma_{t}^{2}=\alpha_{{}^{\circ}}+\alpha_{1}e_{t-1}^{2}+\cdots +\alpha_{q}e_{t-q}^{2}+\beta_{1}\sigma_{t-1}^{2}+\cdots +\beta_{p}\sigma_{t-p}^{2}$$ (3)

Where α° > 0, term is the constant in the model, which represents the long-run average of the conditional variance while

$$\alpha_{i}\geq 0\;for\;i=1,\ldots .,q;\;\beta_{i}\geq 0\;for\;i=1,\ldots .,p$$

are imposed to guarantee that the conditional variance is non-negative.

#### Exponential Generalized Autoregressive Conditional Heteroscedasticity (EGARCH) model

The exponential GARCH can be written as follows:

$$\varepsilon_{t}=\sigma_{t}z_{t}$$ (4)

$$ln\sigma^{2}=\omega +\sum_{i=1}^{p}\alpha_{i}\varepsilon_{t-i}^{2}+\sum_{j=1}^{q}\beta_{j}ln\sigma_{t-j}^{2}$$ (5)

Because of the log variance, this model differs from the GARCH structure which captures asymmetrical consequences of shocks. In the financial literature, the following specification has also been used (Ali 2013) [41].


$$\varepsilon_{t}=\sigma_{t}z_{t}$$ (6)

$$ln\sigma^{2}=\omega +\alpha_{i}\varepsilon_{t-i}^{2}+\sum_{j=1}^{q}\lambda_{j}\ln\left(\sigma_{t-j}^{2}\right)+\sum_{i=1}^{p}\gamma_{i}(\frac{\left|\varepsilon_{t-i}\right|}{\sigma_{t-i}}-\sqrt{\frac{2}{\pi }})$$ (7)

Where *z*_*t*_ represents the standardized residual at time t, *α*_*i*_, *λ*_*j*_, *γ*_*t*_ are coefficients of lagged squared residuals, lagged logarithms of the squared conditional standard deviations and standardized residuals.

### Deep learning artificial intelligence

#### Artificial Neural Network (ANNs)

ANNs are the cutting-edge machine-learning algorithm. Features of ANN are its inherently non-linearity, data-driven and self-adaptive approach and universal approximate function [42].

The output of the ANN model is computed using the following mathematical expression:

$$y_{t}=a_{{}^{\circ}}+\sum_{j=1}^{q}a_{j}g\left(\beta_{{}^{\circ}j}+\sum_{i=1}^{p}\beta_{ij}y_{t-i}n\right)+\varepsilon_{t,}$$ (8)

**Fig 1:**
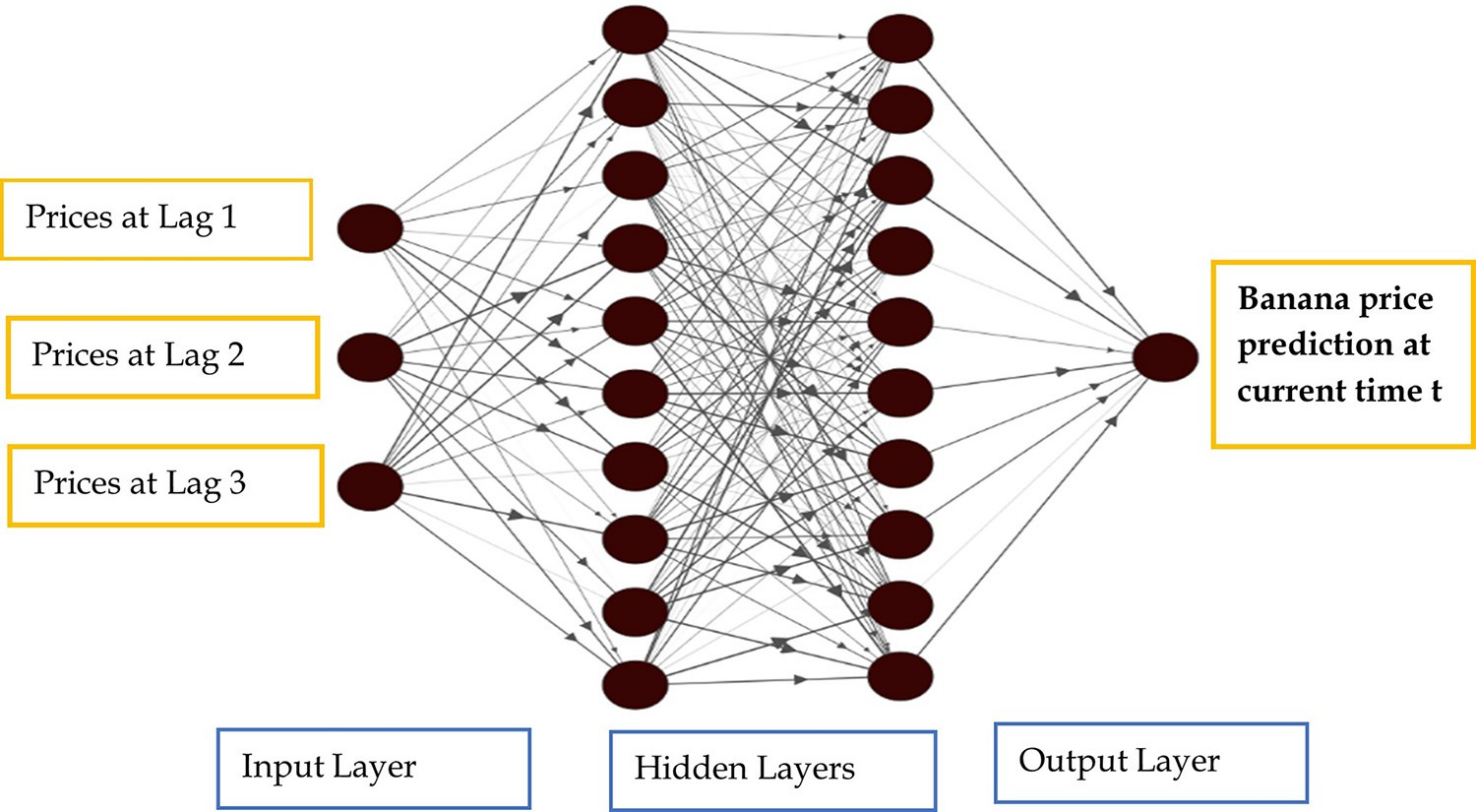
Architecture of Artificial Neural Network (ANN). The diagram was created using NN-SVG (Lenail, 2015) [43].

Here *y*_*t−i*_ (i = 1, 2,…, p) are the *p* inputs and *y*_*t*_ is the output. The integers *p*, *q* are the number of input and hidden nodes respectively. *a*_*j*_ (j = 0,1,2,………q) and *β*_*ij*_ (i = 0,1,2,………q) are the connection weights and *ε*_*t*_ is the random shock. *a*_*j*_ and *β*_°*j*_ are the bias terms and *g(x)* is the nonlinear activation function [42]. The architecture of feed forward neural network is described in Fig 1.

#### Recurrent Neural Networks (RNNs)

**Fig 2:**
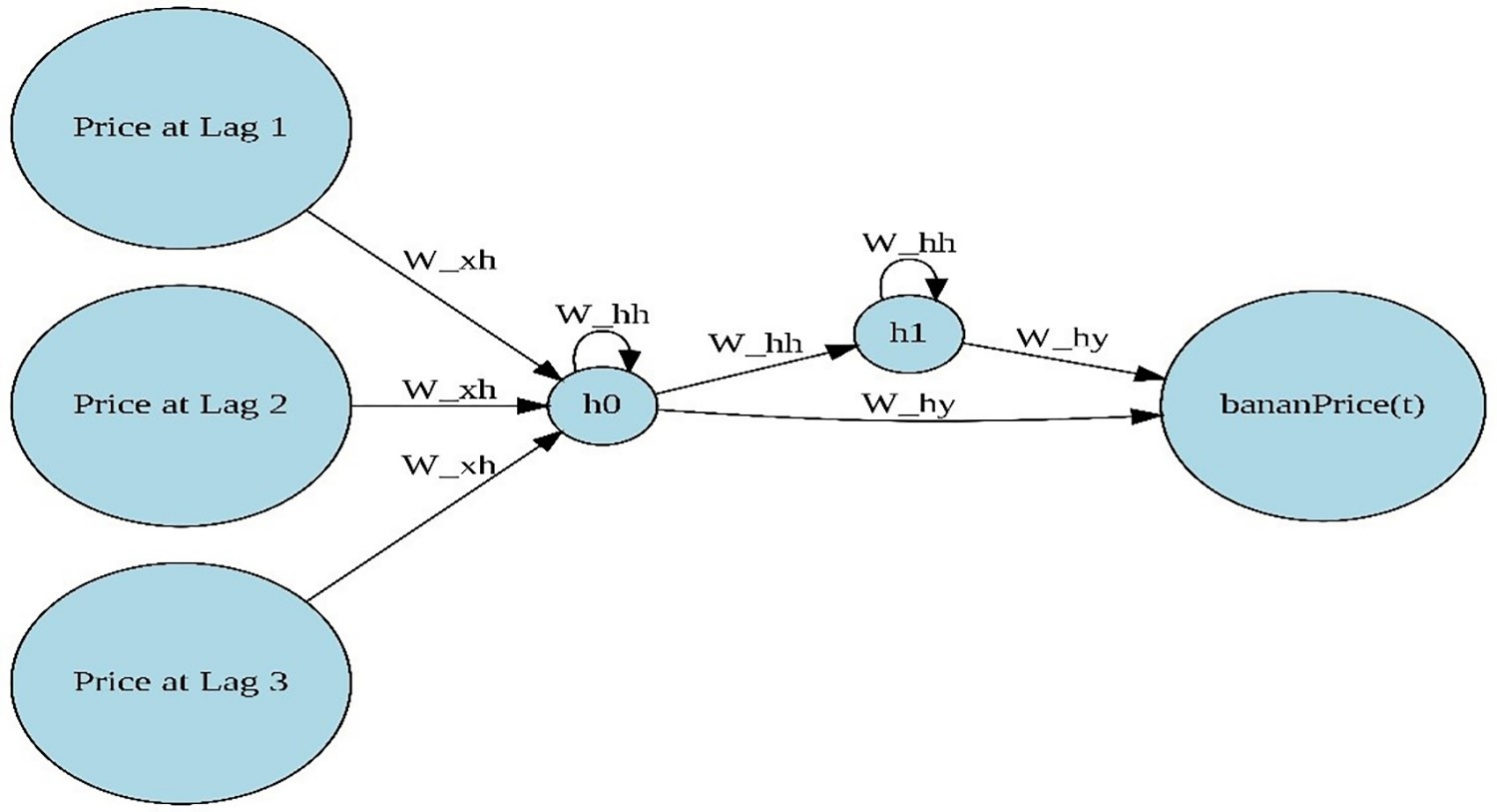
Architecture of Recurrent Neural Network (RNN). The diagram was created using PyTorch and graphviz library [44–45].

The RNN’s goal is to process sequences of data. Unlike the traditional neural network architecture, which connects the input layer to the hidden layer to the output layer in a fully connected manner with no inter-layer connections, this ordinary neural network is inadequate for many problems. RNN, on the other hand, is referred to as a recurrent neural network since the current output of a sequence is influenced by the previous output as given in Fig 2. This implies that the hidden layer nodes are no longer isolated but rather interconnected and the input to the hidden layer includes not only the input layer’s output but also the previous hidden layer’s output.

RNNs are a type of artificial neural network that is designed to work with time series data that contains sequences. It has the concept of "memory," which stores the information of previous inputs in order to get the next output [46]. A feedback loop is present in a basic RNN as illustrated in Fig 2. The equation of RNN can be expressed as:

$$h_{t}=f(W_{xh}X_{t}+W_{hh}H_{t-1}$$ (9)

$$O_{t}=f(W_{hy}S_{t})$$ (10)

*X*_*t*_ is input at time t, *H*_*t*−1_ is state at time *t-1*, *W*_*xh*_, *W*_*hh*_ *and W*_*hy*_ are weight matrices for input, hidden and output layers.

#### Accuracy measures

All the models were evaluated based on the Root Mean Square Error (RMSE) [47], Mean Absolute Percent Error (MAPE) [47], Mean Absolute Scaled Error (MASE) [48], Symmetric Mean Absolute Percentage Error (SMAPE) [49] and Mean Directional Accuracy (MDA) [50] having details mentioned in S1 File.

## Result and discussion

### Result

#### Data pre-processing

Data pre-processing and statistical investigation for prices of banana in Gujarat was done before using it for forecasting models. The dataset of banana prices in Gujarat has mean Rs. 806.76/quintal while 2nd quartile Rs. 800.00/quintal, reflects slight deviation from non-normality. Also, banana price ranges from minimum of 183.33 (Rs. /quintal) to maximum of 1533.33 (Rs. /quintal) indicating high variation exist among the dataset. Furthermore, data exhibit high degree of asymmetry and is characterized by a leptokurtic shape Table 1.

**Table 1:** Banana price dataset description.

*Parameter*	Rs./q
*Number of observations*	132.00
*Mean*	806.76
*Standard deviation*	384.83
*Minimum value*	183.33
*1^st^ quartile*	379.57
*2^nd^ quartile*	800.00
*3^rd^ quartile*	1200.00
*Maximum value*	1533.33
*Skewness*	-0.15
*Kurtosis*	-1.22

**Fig 3:**
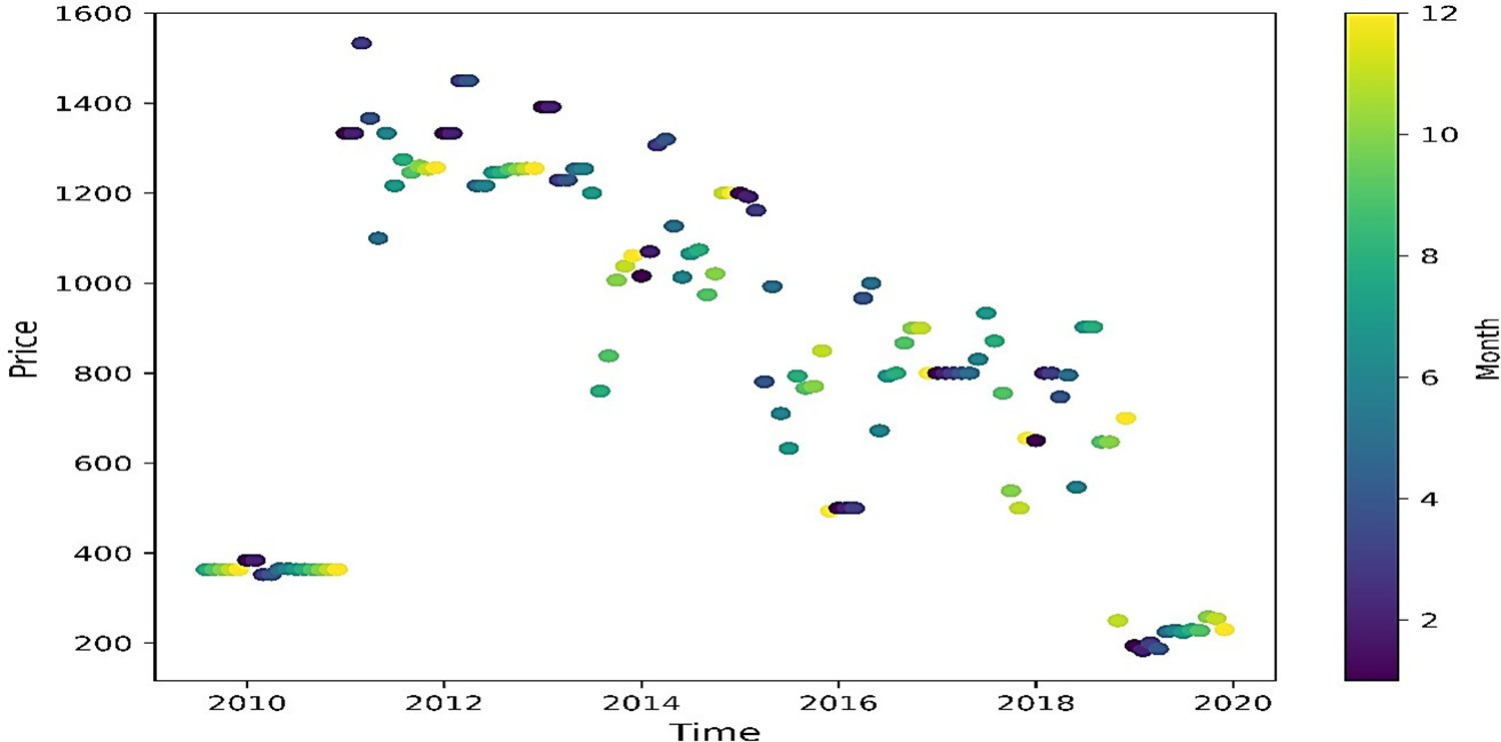
Time series scattered plot for banana prices in Gujarat.

Time series price movement over the period of January, 2009 to December, 2019, shown in Fig 3, signifying high prices mostly occur in the month of Feb–April (colour palette 2 to 4) every year, which reflects seasonal impact.

**Fig 4:**
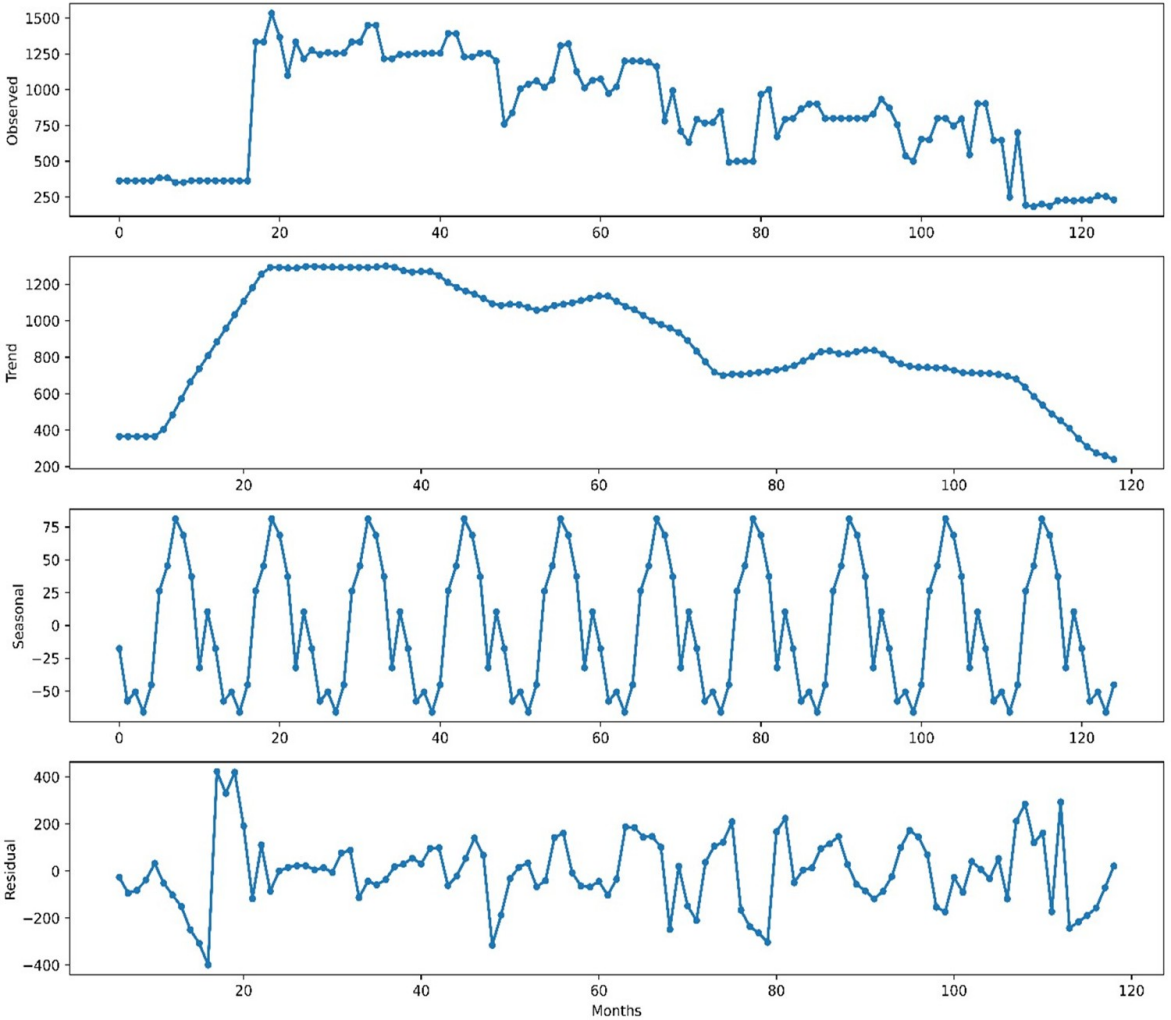
Time series decomposition of banana prices in Gujarat.

The first subplot of Fig 4 reveals the original time series graph depicting the fluctuations in banana prices in Gujarat. It highlights a significant price surge from 2009 to 2011, followed by a decline between 2018 and 2019. These fluctuations introduce non-linearity into the dataset during that specific period. Further, the time series data of banana prices is decomposed into trend, seasonal and residual components using additive seasonal decomposition techniques in the subplot 2^nd^, 3^rd^ and 4^th^ of Fig 4. The 2^nd^ subplot is basically reflecting the long-time trend of banana prices with some shift in the initial as well at the end year and that is because of a lot of missing data in the initial year while the distorted price in 2019, as mentioned on the Agmarknet website.

Additionally, the 3rd subplot reveals a noticeable pattern of seasonality, specifically in the months of February through April, as well as during September to October. Last residual subplot is basically showing variations in the prices of banana which is quite high for the period of 2009–2011 and 2018–19.

**Fig 5:**
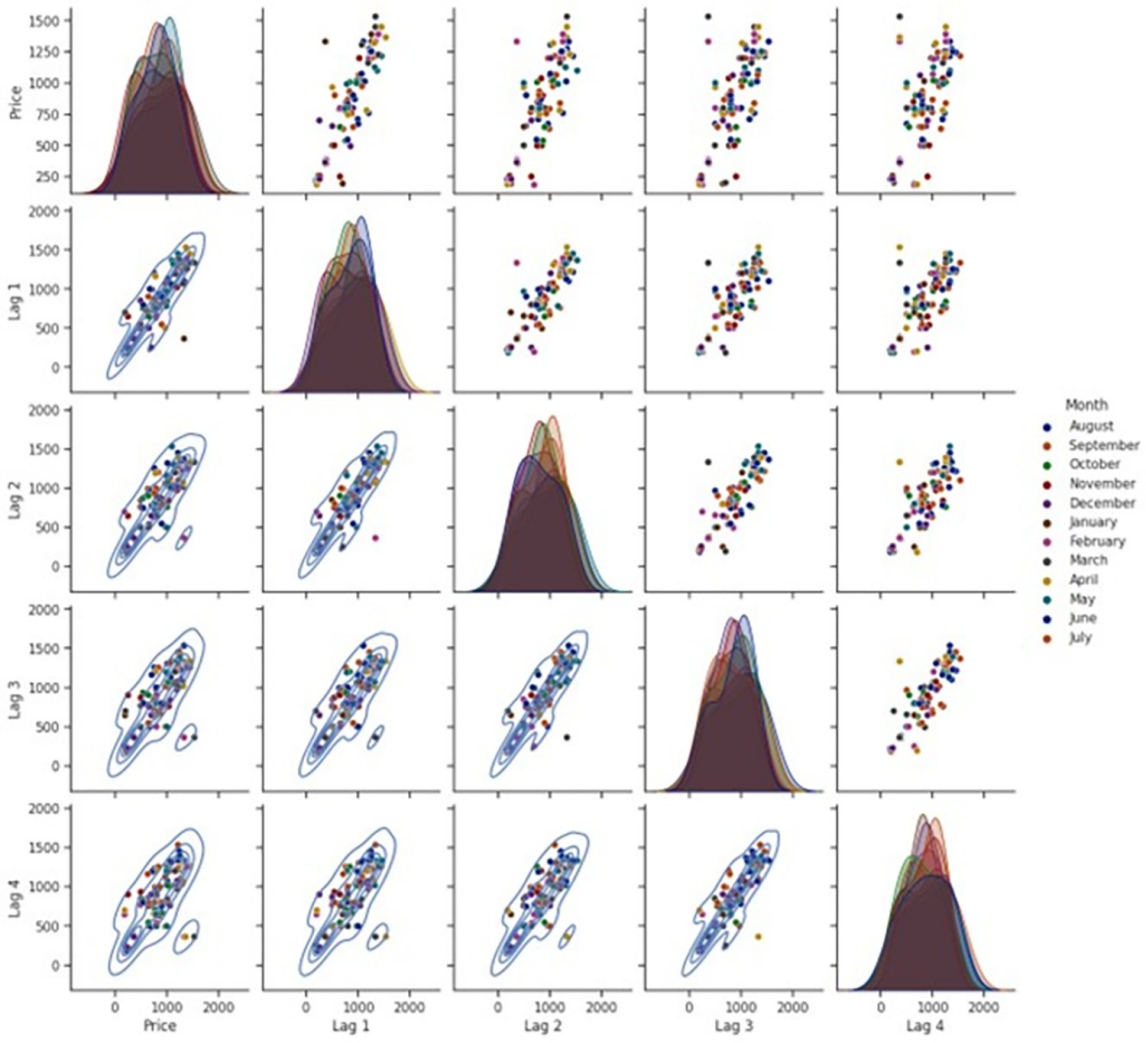
Pair-plot of multiple lag prices of banana in Gujarat.

Fig 5 is reflecting the strength of relationships among multiple lag prices of banana.

The upper triangle of pair plot is indicating that previous four month’s prices are highly correlated with current prices indicating serial correlation and non-randomness among the price with its multiple lags. In the diagonal plot, kernel density estimates (KDE, univariate) are plotted for current price as well for previous month (Lag 1 to Lag 4) indicating that distribution of data is not normal while in the lower triangle of pair plot, kernel density estimates (KDE, Multivariate) of prices are represented as a contour plot depicting the relationship of the non-normal probability distribution between those two variables.

**Fig 6:**
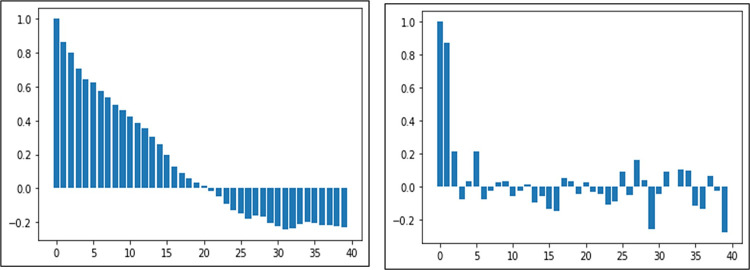
ACF& PACF lags of time series data for banana price in Gujarat.

The plots of Auto correlation function (ACF) and Partial Auto correlation function (PACF) were generated in Fig 6, for determining the stationarity of time series data of banana prices in Gujarat, which confirms that ACF of the price lag reflect a slow decline, indicating the presence of non-stationarity.

This is supported by the non-significant p value of the Augmented Dickey Fuller (ADF) [43] test statistics in Table 2 indicating acceptance of null hypothesis. Therefore, differencing is required to make it stationary which becomes stationary after one differencing.

**Table 2:** ADF test for banana prices of Rajpipla market.

ADF test value	t-statistic	Probability
**Original dataset**	-1.90	0.33
**After one differencing**	-7.63	<0.01

The linearity of fitted model of time series data was judged based on Lagrange’s Multiplier test. Table 3 displays the test statistic values at various lags, indicating a high level of significance at a 5% level of significance. This implies a robust presence of volatility impact on the banana price series.

**Table 3:** Lagrange Multiplier Test of ARIMA model.

Order	LM test statistic	p-value
**4**	312.9	<0.01
**8**	137.2	<0.01
**12**	75.2	<0.01
**16**	46.8	<0.01

This description suggests that data exhibits non-normality, strong autocorrelation among lagged prices, seasonality, a large dispersion potentially caused by shifts, non-stationarity, slight skewness and leptokurtic distribution. These characteristics must be considered during the development of any models.

#### Model building

Table 4 shows the train-test split of the datasets as well as the period of forecasting for Banana. The model’s robustness was assessed by developing it on training data points, as specified in Table 4. Subsequently, the performance of model was examined on testing data points.

**Table 4:** Details of price data set.

Crop	Year	Month	Total data points	Training & validation data points	Testing data points
**Banana**	2009 to 2019 (11 years)	12	132	**108** [80%] & **12** [10%]	12[10%]
**Forecasting period**		Jan. 2020 to Dec.2020			

The forecasting period for the model was from January 2020 to December 2020, considering data up to the year 2019. The generated forecast for this period was compared against the actual modal prices that were made available on the website during the completion of the study. The simulation procedure was used to assess the reliability of the model.

#### Model performance

The forecasting model like ARIMA, SARIMA, G/ARCH, ANN and RNN as described in methodology sections have been fitted for the training data under consideration. Selection of different model/architecture were solely based on lowest accuracy measures like MSE, RMSE, MAPE, SMAPE and MASE generated on testing data set was considered and are mentioned in Table 5. It shows that among the five models examined, the Recurrent Neural Network (RNN) model has the lowest absolute percentage error (MAPE) of 9.58, indicating its superior suitability for the problem. Additionally, the RNN model exhibits an impressive Mean Absolute Scaled Error (MASE) of 0.12, which highlights its effectiveness in improving accuracy by almost 10 times when compared to the Naïve model. In contrast, the ARIMA, SARIMA and ARCH models demonstrate poor fit, with their respective MASE values exceeding 1. The RNN model outperforms its counterparts and provides the most accurate predictions for the test dataset.

**Table 5:** Accuracy measures of models on test data set of banana price in Gujarat.

Model/performance measures	ARIMA	SARIMA	GARCH	ANN	RNN
**MSE**	44007.7	43614.2	29549.6	13686.7	7157.16
**RMSE**	209.78	208.84	171.9	116.99	84.6
**MAPE**	65.79	74.13	60.91	16.56	9.58
**SMAPE**	30.14	37.80	32.49	11.12	7.43
**MASE**	2.44	12.37	7.78	0.58	0.12
**MDA**	0.55	0.56	0.46	1.00	1.74

**Fig 7:**
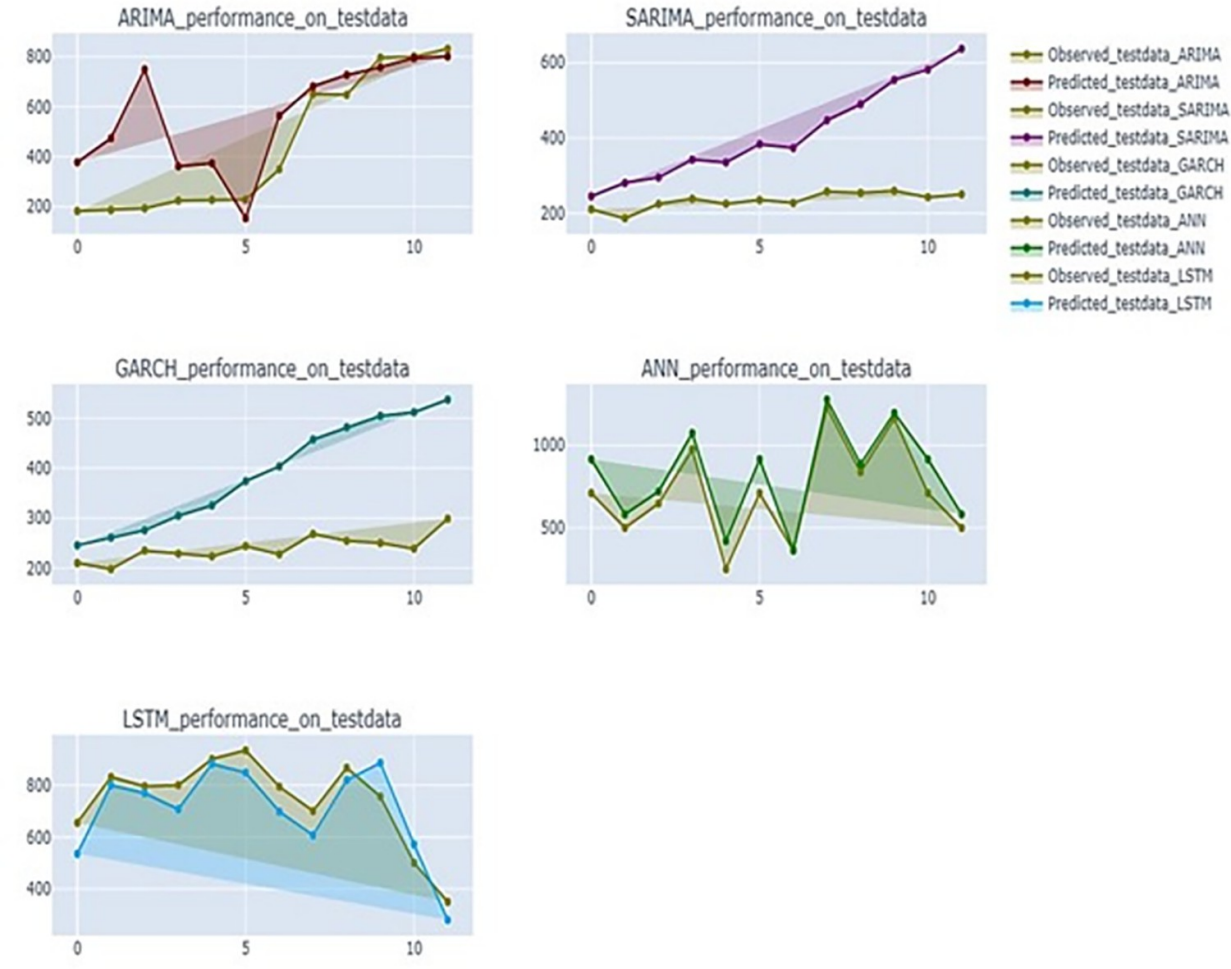
Performance of time series models for banana price on test dataset.

**Fig 8:**
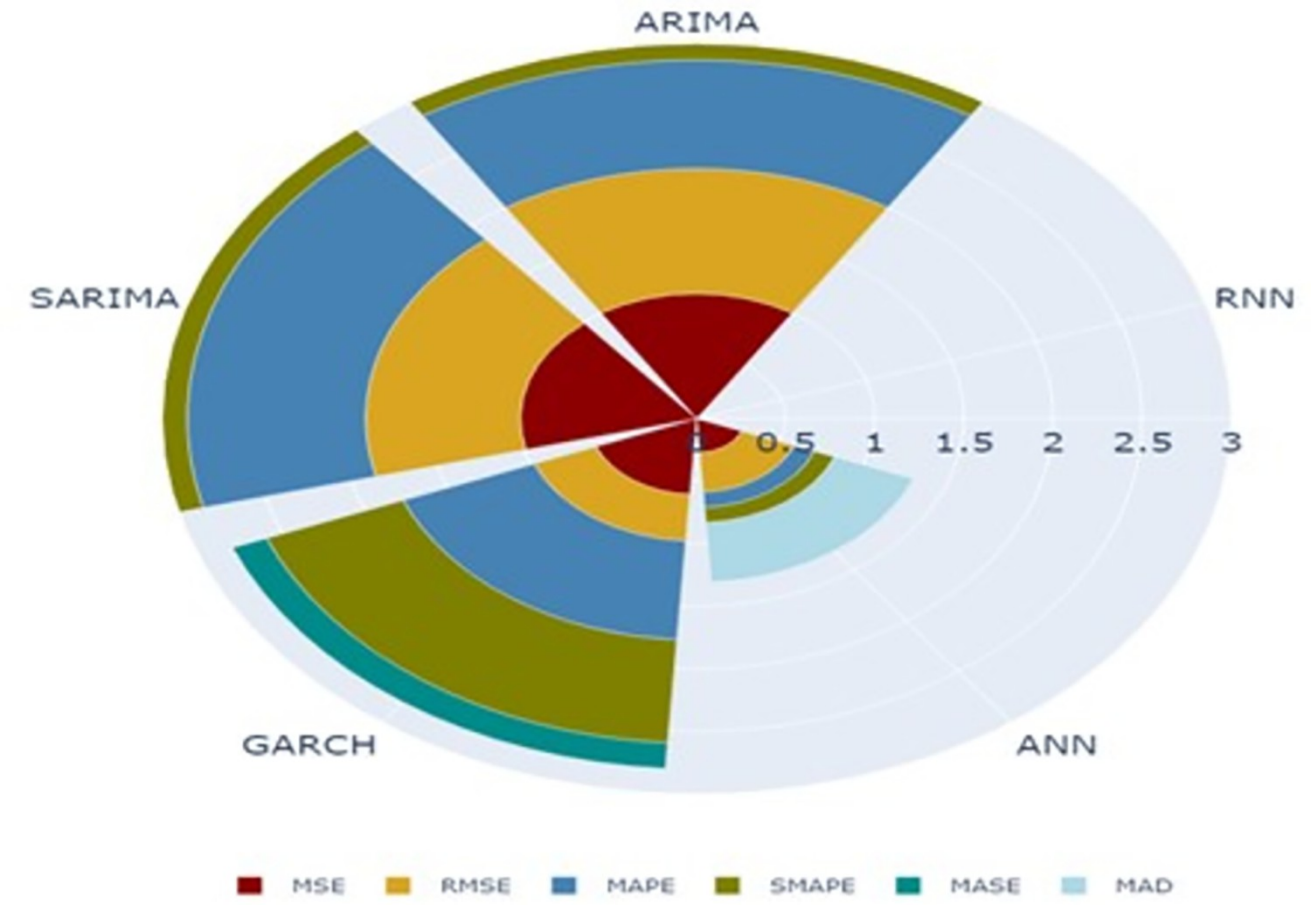
Polar chart of accuracy measures on testing data of time series models for banana prices in Gujarat.

In Fig 7, the predicted and observed values of the test data set were plotted for each model. The graph depicted in Fig 8 provides insight into the predictive capability of RNN model over other models on test data. It presents a polar chart illustrating the scaled accuracy measures of five models on test data of banana prices in Gujarat. The RNN model surpasses remaining five models across various error metrics, including MSE, RMSE, MAPE, SMAPE and MASE.

Moreover, the fitted models were leveraged to make forecast for the upcoming 12-month period for the year 2020, encompassing the months that have been adversely impacted by the COVID pandemic. The actual reported value for the given time interval was compared with the projected value derived from a range of time series models. In situations where other methodologies faltered, recurrent neural networks (RNNs) outperformed others in predicting the prices of bananas.

Table 6 presents a comprehensive breakdown of the accuracy measures for each model during the forecast period. It is evident that RMSE, MAPE, SMAPE and MASE decrease significantly as the models move from ARIMA to RNN, over the forecasting time span. Furthermore, mean directional accuracy (MAD) demonstrates that RNN outperforms other models in predicting the direction accuracy.

**Table 6:** Accuracy measures of models on forecast data of banana price in Gujarat.

Model/performance measures	ARIMA	SARIMA	GARCH	ANN	RNN
MSE	395539.10	309982.60	80782.06	64153.66	6256.16
RMSE	628.919	556.76	284.22	253.28	79.09
MAPE	68.84	58.211	25.73	13.46	4.77
SMAPE	59.96	48.80	17.42	10.23	2.81
MASE	5.77	4.98	2.08	1.40	0.28
MDA	0.45	0.45	0.71	0.80	0.90

**Fig 9:**
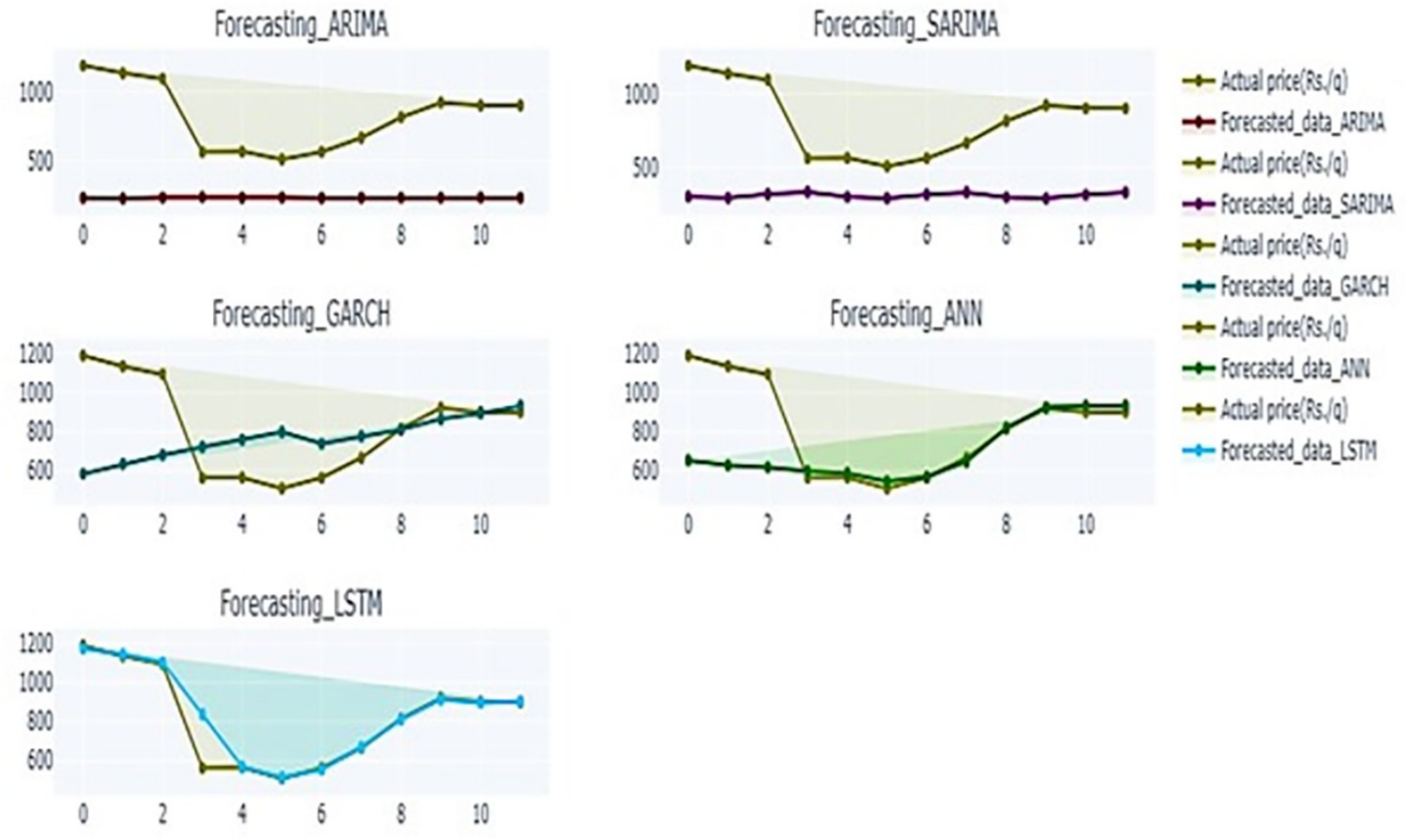
Performance of time series models for banana price for forecasted period.

**Fig 10:**
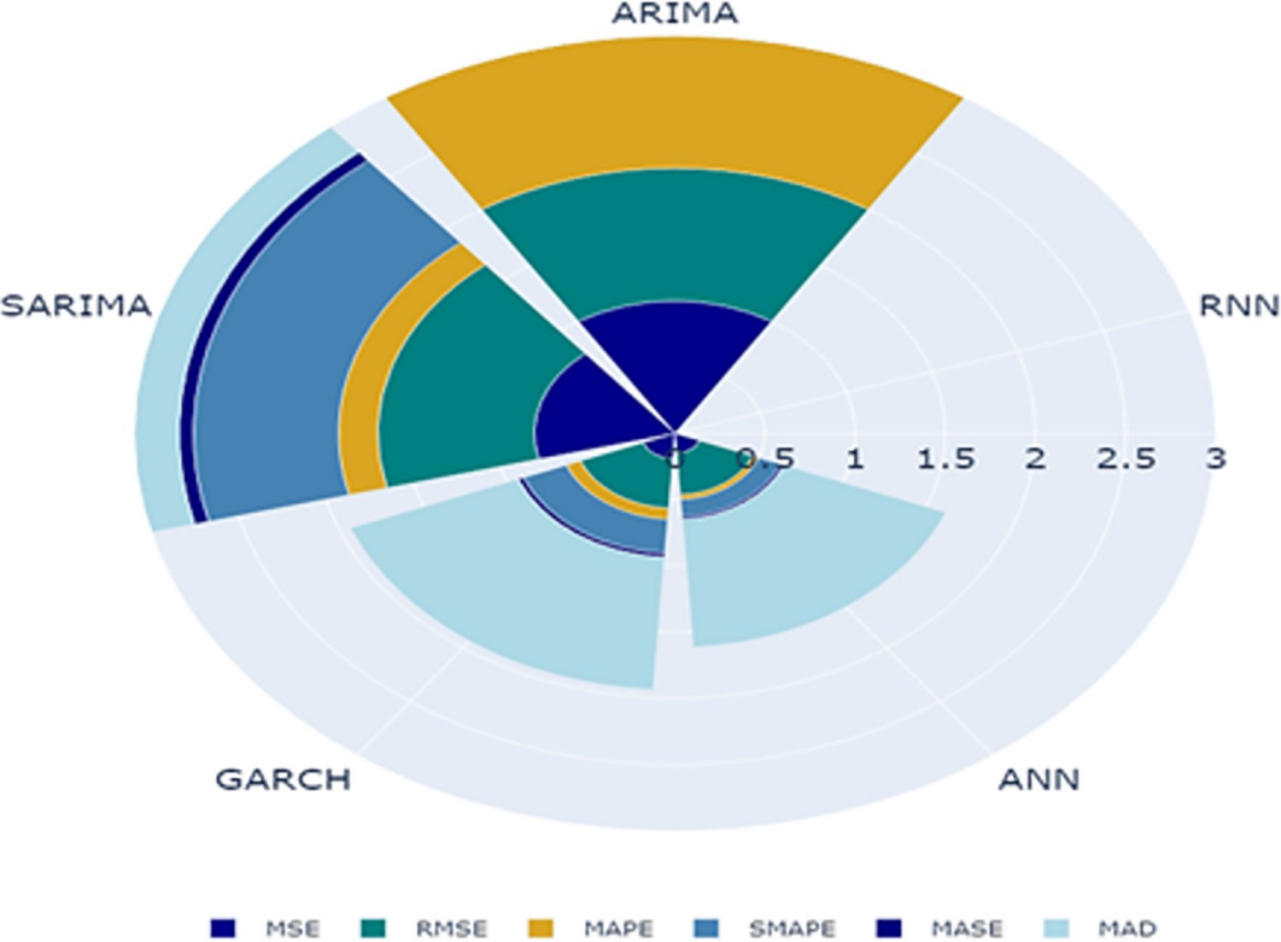
Polar chart of accuracy measures on forecasted data of time series models for banana prices in Gujarat.

Fig 9 presents a comparison of the forecasting performance of various models over the period of January 2020 to December 2020. It shows how closely each model’s forecast aligns with its corresponding actual reported value. The figure serves as a reliability check of model during forecast period from January to December 2020 which is basically the COVID 19 period. Additionally, a polar chart, displayed in Fig 10, shows scaled measures for model accuracy, revealing negligible error and hence better accuracy for RNN across all five measures. Furthermore, a paired t-test was performed to determine the statistical significance of the difference between the forecasted price generated by the model and the actual prices during the forecast period. Table 7 shows that there was no significant difference between the forecasts of the RNN, while the ARIMA and SARIMA showed significant differences, indicating their poor fit.

**Table 7:** Significance check of forecast of models on banana price in Gujarat.

*Month & Year*	Actual price(₹/q)	Forecasted value (₹/q)				
		RNN	ANN	G/ARCH	SARIMA	ARIMA
*Jan-20*	1187.68	1172.21	654.15	585.91	305.94	233.5
*Feb-20*	1133.15	1142.67	628.22	636.3	297.67	227.77
*Mar-20*	1092.35	1100.98	618.19	681.69	322.6	235.12
*Apr-20*	564.41	837.2	598.41	722.58	338.45	238.02
*May-20*	566.66	568.71	588.25	759.42	303.71	234.1
*Jun-20*	510.5	515.86	545.4	798.96	295.44	235.67
*Jul-20*	564.77	554.99	570.23	737.83	320.37	233.15
*Aug-20*	666.42	671.19	647.02	776.7	336.22	234.2
*Sep-20*	815.25	810.86	818.57	810.57	301.48	235.03
*Oct-20*	920.65	913.1	925.31	864.44	293.21	234.3
*Nov-20*	900	896.02	932.16	893.3	318.14	235.8
*Dec-20*	900	903.43	933.08	932.17	333.99	234.33
***p value** of t test (b/w Forecast & Actual)*		**0.37**	**0.12**	**0.55**	**0.00**	**0.00**

#### Model fit parameters

This section describes the parameters and hyperparameters of the RNN model, which was identified as the most suitable model among all other models *i*.*e*. ARIMA, SARIMA, ARCH and ANN. Table 8 presents the details of the optimal RNN model’s architecture and its parameters.

**Table 8:** Tuning parameters of RNN.

Parameters			Other hyperparmeters	
Number of input		3	Learning Rate (α)	0.0001
timesteps		1	Regularistaion Method	L1
Features		1	Regularistaion Coefficient (λ)	0.001
Neorns in layers	Hidden	10	Drop out	0.2
	Output	1	Batch size	2
Activation function in layers	Hidden	ReLU	Epochs	40
	Output	Linear	Optimiser	Adaptive Moment Estimation

The proposed RNN architecture as mentioned in Table 8, aims to address overfitting through a combination of parameter choices and regularization techniques. The model comprises an input layer with 3 input values (previous lag value of price), a hidden layer with 10 neurons and an output layer with a single neuron. The ReLU activation function is employed in the hidden layer, promoting non-linearity and feature representation while a linear activation function for output layer is used to allow direct prediction of continuous values. To prevent overfitting, L1 regularization is applied with a coefficient of 0.001, encouraging sparse weight values and reducing dependence on specific features. Additionally, dropout is implemented with a rate of 0.2, randomly deactivating hidden units during training to reduce dependence on individual neurons.

## Discussion

The findings indicate that the ARCH/GARCH statistical models performed better than the ARIMA and SARIMA models. One suggestion put forth by certain academic experts is to combine the ARIMA model with the GARCH model to overcome the limitations of linear models [51]. However, even with this approach, there remain various factors contributing to volatility that cannot be effectively addressed by these conventional models alone. In such cases, the utilization of advanced machine learning models could prove invaluable in capturing and addressing the full spectrum of variables affecting volatility.

Nonetheless, statistical models ARIMA/SARIMA and ARCH/GARCH were unable to compete with the neural network models, which produced more accurate results. This discovery is consistent with the study conducted by [52], in which they concluded that the artificial neural network (ANN) model is a significant substitute for theoretical models in anticipating the rainfall-runoff dataset. Additionally, out of two neural network architecture, RNN performed better than ANN. Non-linearity as well as time sequence data, give Recurrent Neural Network, more power. The same is supported by the finding in [53] where accuracy of deep-learning RNN methods are better and more accurate than ANN while simulating the streamflow of reservoir. However, some previous studies have found contradictory results. For instance, a study by [54] found ARIMA outperformed ANN in predicting stock prices while in [55] GARCH models outperformed deep learning models in predicting the volatility of the Shanghai Composite Index. Thus, methods like S/ARIMA, G/ARCH and ANN have proven to be valuable initial approaches, it is crucial to acknowledge their inherent limitations due to their data-specific nature and supplement them with more comprehensive techniques having capabilities to deal with dynamic data when necessary.

## Conclusion

After analysing various models to anticipate banana prices in Gujarat for the COVID period from January 2020 to December 2020, our study found that the Recurrent Neural Network (RNN) outperformed all other models in terms of RMSE, MAPE, SMAPE, MASE and MAD values, showing its ability to handle unexpected events and their impact on future prices. Our results suggest that the RNN model can aid policymakers in improving their decision-making processes, leading to increased profitability. The practical applications of this research findings include the development of tools and applications that farmers, traders and end-users can use to access the forecasted prices of bananas in Gujarat. Policymakers can also use the results to address the challenges that farmers and traders face in the market.

However, our study also has limitations like it only considers its own price lag value and haven’t incorporate other essential factors such as weather, commodity arrival, demand & supply, government export-import policies and commodity diversity into the forecast. As a result, future research should explore different deep learning architectures which incorporate these variables to improve prediction accuracy. Addressing these limitations would lead to more comprehensive and nuanced conclusions in this study, along with practical applications for farmers, traders, and end-users in the horticultural commodity market.

## Supporting information

S1 File Accuracy measures of model. (PDF)
