# Supplementary material for: Recurrent neural network architecture for forecasting banana prices in Gujarat, India
Source: PLoS One. 2023 Jun 15;18(6):e0275702. doi: 10.1371/journal.pone.0275702 (PMC10270605; doi:10.1371/journal.pone.0275702)
Supplement: S1 File — (PDF) [file pone.0275702.s001.pdf]

Accuracy Measures of Model

All the models were evaluated based on the following accuracy metrics:

1. **Root Mean Square Error (RMSE)**

Root Mean Square Error (RMSE) is defined as the square root of mean square error which is sum of squared errors divided by total numbers of observations. The formula for RMSE is:

$$RMSE = \sqrt{\frac{1}{n}\sum_{t=1}^{n}(F_t - A_t)^2} \quad \text{……………………..} (1)$$

2. **Mean Absolute Percent Error (MAPE)**

In statistics, the Mean Absolute Percent Error (MAPE) is a gauge of a method's accuracy in producing fitted time series data. It is commonly expressed as a percentage and is calculated using the formula:

$$MAPE = \frac{1}{n}\sum_{t=1}^{n}\left|\frac{(F_t - A_t)}{A_t}\right| \times 100 \quad \text{………………..} (2)$$

3. **Mean Absolute Scaled Error (MASE)**

Mean Absolute Scaled Error (MASE) is a measure for used to measure the effectiveness of forecasts generated through the model by comparing it with naïve forecast. It is commonly expressed by using the formula:

$$MASE = \frac{\frac{1}{n}\sum_{t=1}^{n}|F_t - A_t|}{\frac{1}{n-1}\sum_{t=2}^{n}|A_t - A_{t-1}|} \quad \text{……………………..} (3)$$

4. **Symmetric Mean Absolute Percentage Error (SMAPE)**

Symmetric Mean Absolute Percentage Error (SMAPE) another accuracy metric which is expressed by using the formula:

$$SMAPE = \frac{1}{n}\sum_{i=1}^{n}\frac{|F_t - A_t|}{F_t + A_t/2} \quad \text{…………………..} (4)$$

5. **Mean Directional Accuracy (MDA)**

It measures the effectiveness of a model in correctly determining the direction of an outcome relative to its actual outcome.

$$MDA = \frac{1}{n}\sum_t 1_{sgn}(A_t - A_{t-1}) == sgn(F_t - A_{t-1}) \quad \text{……….} (5)$$

Where F_t is a forecasted value for time t, A_t is the actual value for time t, A_{t-1} is previous value at time t-1 and n is the total number of forecasts.
